# A58 UTILIZATION OF HYPOXIA-TOLERANT ORGANISMS AS A MODEL IN THE STUDY OF ISCHEMIC-REPERFUSION INJURY IN TRANSPLANT HEPATOLOGY

**DOI:** 10.1093/jcag/gwad061.058

**Published:** 2024-02-14

**Authors:** N Hossein-Javaheri, L Buck

**Affiliations:** Internal Medicine, University at Buffalo, Buffalo, NY; University of Toronto, Toronto, ON, Canada

## Abstract

**Background:**

Ischemic-reperfusion injury (IRI) is a barrier to successful liver transplantation. This complex process is initiated by an episode of hypoxia and decreased adenosine triphosphate (ATP) production. Mammalian hepatocytes are susceptible to prolonged hypoxia and may experience irreversible damage with ATP depletion. Yet, facultative anaerobes have developed physiological hepatoprotective strategies to tolerate hypoxic stress. The painted turtle (*Chrysemys picta belli*) and the common goldfish (*Carassius auratus*) are able to survive under severe hypoxia for weeks to months.

**Aims:**

Introducing hypoxia-tolerant organisms as a suitable animal model for studying IRI in transplant hepatology.

**Methods:**

A comprehensive search of PubMed, OVID, CINAHL, and Cochrane databases up to May 2023 was conducted to identify all studies reporting experimental evidence and hepatoprotective pathways in hypoxia-tolerant organisms (HTOs). Each article was qualitatively assessed. The primary focus was on cell death, intracellular ion gradient, mitochondrial function, and reactive oxygen species (ROS) with hypoxia and IRI.

**Results:**

HTOs have increased glycogen storage with a greater ATP yield from anaerobic metabolism (glycolysis). Approximately 15-30% of liver mass in HTOs is composed of glycogen which is massive compared to 5-6% in mammals. Turtle isolated hepatocytes can tolerate 10 hours of anoxia by reducing cellular metabolic demand by 90% through decreased protein synthesis and ion channel activity. Goldfish hepatocytes can tolerate 6 hours of anoxia via similar strategies. In mammals, mitochondria undergo depolarization, Ca2+ efflux, cellular swelling, and apoptosis with anoxia. Yet, a depolarized mitochondrial membrane potential is regulated to provide cellular protection in HTOs. The ability to preserve mitochondrial function and electrochemical gradients with oxygen lack is an important contributor to cellular survival. Even in mammalian hepatocytes, if the mitochondrial integrity is maintained, cells are better able to tolerate ischemic insults. Finally, formation of radicals with reperfusion leads to hepatocyte apoptosis and necrosis in the mammalian model. Meanwhile, regulated ROS levels in turtles and goldfish cells, prevent cell death and IRI.

**Conclusions:**

IRI is of significant interest in transplant hepatology. Although extremely valuable, the traditional mammalian models are vulnerable to hypoxia. Meanwhile, HTOs have undergone years of adaptation with thousands of genes dedicated to hypoxia tolerance. Utilizing these organisms can provide a broader understanding of IRI and avoid irreversible tissue damage in transplant hepatology.

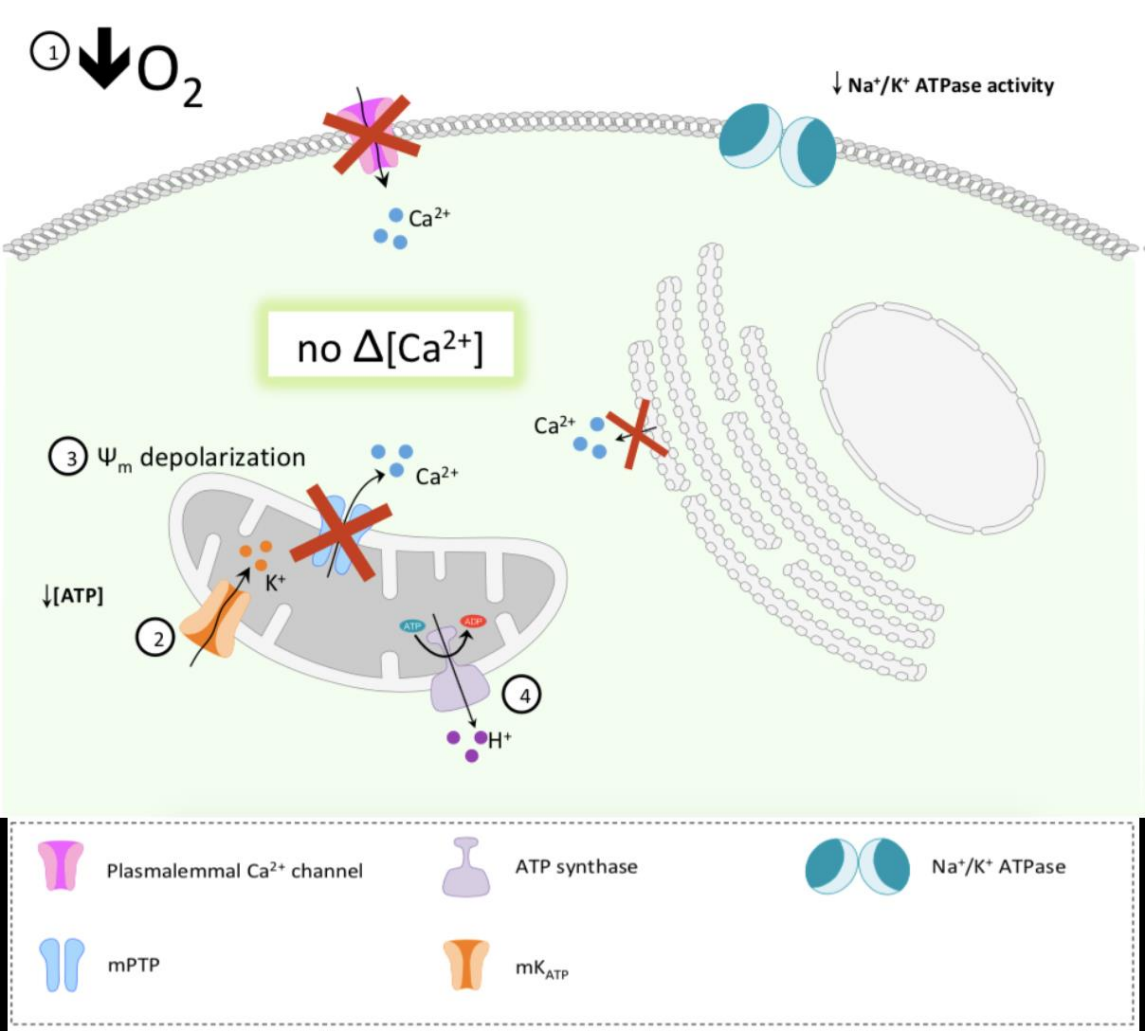

**Funding Agencies:**

CIHR

